# Satisfied or unaware? Racial differences in perceived weight status

**DOI:** 10.1186/1479-5868-3-40

**Published:** 2006-11-12

**Authors:** Gary G Bennett, Kathleen Y Wolin

**Affiliations:** 1 Department of Society, Human Development and Health, Harvard School of Public Health, Boston, USA; 2 Center for Community-Based Research, Dana-Farber Cancer Institute, Boston, USA; 3 Department of Preventive Medicine, Feinberg School of Medicine, Northwestern University, Chicago, USA

## Abstract

**Background:**

Obesity is disproportionately prevalent among many racial/ethnic minority communities. The efficacy of weight control efforts in these groups may depend on individual's ability to accurately perceive their weight status. We examined whether racial/ethnic differences exist in weight status misperception among overweight adults.

**Methods:**

Nationally-representative data from the National Health and Nutrition Examination Survey (NHANES) 1999–2002 were examined. Participants included overweight and obese adult men (n = 3115) and women (n = 3437). Weight status misperception was identified among respondents who self-reported being "about the right weight/underweight."

**Results:**

Blacks (OR = 2.06, 95% CI: 1.71, 2.54) were twice as likely and Hispanics (OR = 1.70, 95%CI: 1.33, 2.17) were 70-percent more likely than Whites to misperceive their weight, in models adjusted for age, education, income, marital status, self-reported health, and self-reported medical diagnosis of overweight. Black overweight (OR = 2.03, 95% CI: 1.26, 3.26) and obese (OR = 3.56, 95% CI: 1.57, 8.11) women were considerably more likely to exhibit misperception compared to their White female counterparts. Odds of misperception were higher among overweight Black (OR = 2.20, 95%CI: 1.54, 3.15), Hispanic (OR = 1.89, 95% CI: 1.30, 2.75), and obese Black men (OR = 2.84, 95% CI: 1.54, 5.22), compared to White men.

**Conclusion:**

Weight status misperceptions among the overweight are more common among Blacks, and Hispanic men. The persistence of racial/ethnic differences after adjustment for medical diagnosis of overweight may suggest some resistance to physician weight counseling. Identifying strategies to correct weight status misperceptions status may be necessary to ensure the efficacy of clinical and public health obesity interventions conducted among these groups.

## Introduction

The epidemic of obesity in the US shows no signs of abating – presently, over 65% of the US population are either overweight or obese [1]. Many racial/ethnic populations are disproportionately affected by the condition; non-Hispanic Black women, for example, are more than twice as likely to be obese, compared to non-Hispanic White women[1]. Obesity control efforts conducted among racial/ethnic minority populations may be hindered by numerous sociocultural factors that influence both dietary and physical activity patterns [2]. For example, Blacks appear to have a greater social acceptance of overweight, less body weight dissatisfaction, and higher body weight ideals compared to Whites [2-8]. Additionally, emerging evidence in nationally-representative cohorts suggest that Blacks, and to a lesser extent Hispanics, may have lower rates of perceived overweight compared to Whites [9-11].

Indeed, Kuchler et al [9] reported that overweight Blacks and Hispanics were significantly more likely to perceive themselves to be at average weight, compared to Whites. Inaccurate weight status perceptions have been posited to drive obesity-related behaviors, such as food consumption and intentional physical activity [2,9-11]. Importantly however, much of the data examining perceived weight status were collected prior to the secular shift towards greater U.S. public awareness of the obesity epidemic and its associated comorbidities. A consequence of this heightened awareness has been calls for greater physician diagnosis and counseling for weight loss among overweight and obese patients [12-16]. No previous studies have examined racial/ethnic differences in perceived weight status remain after controlling for self-reported receipt of medical diagnosis of overweight.

Thus, the purpose of the present study was to examine whether racial/ethnic differences exist in perceived weight status among overweight adults (accounting for receipt of medical diagnosis of overweight), using data from the 1999–2002 National Health and Nutrition Examination Survey studies.

## Methods

The National Health and Nutrition Examination Survey (NHANES) is a stratified, multistage probability sample of the civilian non-institutionalized U.S. population [17,18]. NHANES includes over sampling of Mexican-Americans and non-Hispanic Blacks. Approximately 9965 persons aged 2 months to 85 years were studied in NHANES 1999–2000 and 11,309 persons in NHANES 2001–2002. Race/ethnicity was self-reported by participants; we categorized individuals as: non-Hispanic White, non-Hispanic Black, Hispanic, or other. Height and weight were measured using a standardized protocol at a Mobile Examination Center (MEC)[19,20]. Body mass index (BMI) was calculated as kg/m^2^; we utilized standard thresholds to classify individuals as normal/underweight (BMI<25), overweight (25<=BMI<30), or obese (BMI>=30). Covariates included: age (in years); education (categorized in the three levels available in public release data: less than high school, high school diploma, more than a high school degree); income (<$20,000, $20–34,999, $35–54,999, $55,000+); martial status (married/cohabitating, widowed/divorced/separated, never married), self-reported health status (excellent, very good, good, fair, poor; collapsed into three categories due to small numbers), and; self-report of a medical diagnosis of overweight (yes/no). All analyses were limited to individuals aged 18 and older, who were overweight or obese, and did not report being pregnant. Statistical analyses were performed using SUDAAN (version 9, Research Triangle Institute, Research Triangle Park, NC). Sample weights account for differential non-response, non-coverage and intentional over-sampling of some groups. Logistic regression was used to examine the likelihood of misperception overall, by BMI class, and jointly by BMI class and gender.

### Perceived weight

Participants were asked a standard perceived weight question, "Do you consider yourself now to be overweight, underweight, or about the right weight?" [21,22] As all study participants were overweight (or obese), we considered those whose responses placed them in a combined "about the right weight/underweight" category to exhibit "misperception" or "inaccurate" perceptions of their weight status. Through use of this terminology, we make no attempt to suggest that respondents either intentionally or non-intentionally exhibited inaccuracies in their weight perceptions, as our data do not allow for the explicit examination of this question.

## Results

The prevalence of weight status misperception varied by race/ethnicity and gender. Across racial/ethnic categories, men were more likely to misperceive their weight than women (Table 1). Comparing racial/ethnic categories, for both men and women, Blacks were most likely and Whites least likely to misperceive their weight status. For both men and women, across racial/ethnic categories, the prevalence of weight status misperception was higher among overweight, than among obese participants. Among women, the prevalence of misperception was highest among overweight Black women (40.9%) and lowest among obese White women (3.9%). Similarly, among men, misperception rates were highest among overweight Black men (66.4%), and lowest among obese White men (8.9%).

**Table 1:** Prevalence of underestimation by gender and race/ethnicity, NHANES 1999–2002

	Men (n = 3115)			Women (n = 3437)		
	All	Overweight	Obese	All	Overweight	Obese

White	29.5	43.2	8.9	11.3	20.6	3.1
Black	48.8	66.4	26.2	22.3	40.9	11.2
Hispanic	48.0	63.5	16.8	17.7	29.3	6.7
Other	35.6	50.2	13.0	19.3	23.4	10.4

In multivariable analyses adjusting for all covariates, Blacks (OR = 2.06, 95% CI: 1.71, 2.54) were twice as likely, and Hispanics (OR = 1.70, 95% CI: 1.33, 2.17) were 70-percent more likely than Whites to misperceive their weight. When examining overweight and obese participants separately, the association was stronger for both overweight Blacks (OR = 2.20, 95% CI: 1.54, 3.15) and Hispanics (OR = 1.89, 95% CI: 1.30, 2.76) as well as obese Blacks (OR = 2.84, 95% CI: 1.54, 5.22). The association was attenuated and not significant among obese Hispanics (OR = 1.45, 95% CI: 0.69, 3.07).

When examining men and women separately (Table 2), overweight Black women were twice as likely as overweight White women to misperceive their weight (OR = 2.03, 95% CI: 1.26, 3.26). Overweight Hispanic and White women were equally likely to misperceive their weight (OR = 1.34, 95% CI: 0.85, 2.13). Among obese women, Blacks were over three times as likely as White women to misperceive their weight (OR = 3.56, 95% CI: 1.57, 8.11). Obese Hispanic women were twice as likely as obese White women to misperceive their weight, though the difference was not significant (OR = 2.19, 95% CI: 0.96, 5.01).

**Table 2:** Odds of weight status misperception by race/ethnicity, NHANES 1999–2002*

	Men		Women	
	Overweight OR (95% CI)	Obese OR (95% CI)	Overweight OR (95% CI)	Obese OR (95% CI)

White	Ref	Ref	Ref	Ref
Black	2.20 (1.54, 3.15)	2.84 (1.54, 5.22)	2.03 (1.26, 3.26)	3.56 (1.57, 8.11)
Hispanic	1.89 (1.30, 2.75)	1.45 (0.69, 3.07)	1.34 (0.85, 2.13)	2.19 (0.96, 5.01)
Other	1.04 (0.48, 2.23)	1.21 (0.23, 5.27)	1.58 (0.57, 4.35)	3.94 (0.94, 15.53)

* All models adjusted for age, education, income, marital status, self-reported health status, and self-reported medical diagnosis of overweight.

Both overweight Black (OR = 2.20, 95% CI: 1.54, 3.15) and Hispanic (OR = 1.89, 95% CI: 1.30, 2.75) men had higher odds of misperception, compared to White overweight men. However, among obese participants, only Black men had an increased likelihood of weight status misperception (OR = 2.84, 95% CI: 1.54, 5.22), compared to their White counterparts.

## Discussion

The disproportionately elevated and steadily increasing rates of overweight and obesity among racial/ethnic minority communities [1] constitutes a major public health crisis. The major determinants of the obesity epidemic in all populations – dietary and physical activity practices – may be heavily patterned by sociocultural influences, such as the tendency to misperceive one's weight status. In the present study, we found that overweight and obese Blacks, compared to their White counterparts, were disproportionately more likely to categorize themselves as being "about the right" weight.

Overweight Black men and women each had more than a two-fold increased likelihood of perceiving their weight status. Relative to Whites, obese Black adults were even more likely to exhibit weight status misperceptions. These results were independent of sociodemographic characteristics, traditional individual-level measures of socioeconomic position, and self-reported receipt of physician diagnosis of overweight. Our findings highlight the importance of more frequently incorporating sociocultural factors, particularly perceived weight status, into both clinical and public health obesity reduction approaches for Blacks.

Racial/ethnic differences in perceived weight among women have been previously shown; however, most studies have been conducted among relatively small, select samples, including high proportions of college students. Among the few previous studies conducted using nationally-representative data, Paeratakul et al (in the 1994–1996 Continuing Survey of Food Intakes by Individuals and the Diet and Health Knowledge Survey) showed that Whites were 2.3 times more likely than non-Whites to perceive themselves to be overweight. However, the authors failed to disaggregate the non-White category to examine whether Blacks and/or Hispanics had higher odds of inaccurate perceptions. In the NHANES III cohort, Kuchler and Variyam [9]reported that the prevalence of perceived overweight was lower among both overweight and obese Blacks, compared to Whites. Trends for Hispanic respondents in the study were similar to those of Blacks; however the prevalence of misperception among obese Hispanics did not significantly differ from Whites. Kuchler and Variyam's analysis was limited to the presentation of prevalence data; however, this allows us to compare the prevalence of misperception in the NHANES III cohort (1988–1994) to our data in the NHANES 1999–2002 samples. Over a period of time characterized by secular trends for weight gain in the general population, misperception rates among Black in particular, continued to increase among both men and women. Among overweight men, the prevalence of misperception increased in all racial/ethnic groups (White: 8%, Black: 14%, Hispanic: 22%); however, for obese men, misperception rates decreased for both Whites and Hispanics by about 13.5%, while over the same period they increased by over 49% for Blacks. Among women, sizeable increases were seen in the prevalence of perceived normal weight among both White and Black overweight (44% and 52% respectively) and obese (16% and 21% respectively) women. However, among Hispanic women, a 5% decrease in misperceptions was seen for both overweight and obese women.

What might explain the greater potential for overweight Blacks to misperceive their weight status? As has been previously mentioned, considerable research evidence has identified myriad sociocultural influences (e.g. heavier body image ideals, fewer social pressures to lose weight) that might be implicated [2-8]. An unanswered question concerns whether Blacks' tendency to misperceive their weight status is a function of weight satisfaction, or a lack of awareness about the extent of their overweight. Given previous evidence of Blacks' high levels of weight satisfaction, it is possible that responses to the studied perceived weight question may have been biased among those who were more weight satisfied. Alternatively, our findings may highlight a lack of awareness about the clinical thresholds for overweight and obesity; this limited awareness may be influenced by the high prevalence of the conditions among Blacks. We have previously speculated that, rather than considering their membership in BMI-defined categories, some Blacks might rely on social comparison to make judgments about their respective weight status. Given that overweight and obesity among Blacks (particularly among women) has reached nearly normative levels, such social comparison among the overweight might negatively bias individuals' judgments about their respective weight status. Whether this potential mechanism extends to Black men (given their lower absolute levels of overweight and obesity compared to women) is unclear. The mechanisms responsible for Blacks' weight status perceptions may be particularly important to discern, given our finding that misperceptions persisted after adjustment for self-reported physician diagnosis of overweight.

We were surprised to find that racial/ethnic differences going perceived weight remained after adjusting for adjustment for receipt of physician diagnosis of overweight. Physician diagnosis and counseling for weight reduction among the overweight has increasingly been recommended, [12-16] though its frequency remains low [23]. Given that Blacks remained more likely to misperceive their weight after adjustment for the diagnosis variable, we hypothesize that some Black patients might be resistant to physician diagnoses of overweight. Generic messages of a patient's overweight (i.e. those that do not discuss the clinical thresholds of overweight in some detail, provide a description of the health risks associated with overweight and obesity), or non-comprehensive counseling may have little effect, particularly given that many Blacks do not recognize the health consequences of overweight and obesity [11] and may not be readily motivated by weight-related aesthetic concerns. However, as weight status misperception may also protect against eating and body image disorders (which are less common among Blacks), misperception correction strategies should be carefully considered.

Our study has several strengths and extends prior findings in a number of important ways. First, rather than presenting only prevalence data, our focus was to systematically examine sources of racial/ethnic variation in perceived weight status, adjusted for relevant confounders. Our data were collected during the period of time when public awareness of obesity and its associated health consequences increased dramatically. One might have expected the greater public attention to obesity occurring during this period to have enhanced individual's ability to more accurately perceive their weight status. Of course, a number of considerations may limit interpretations drawn from our findings. BMI as a measure of body weight does not incorporate body fat distribution, which may be differentially associated with obesity-related health conditions by race/ethnicity. Furthermore, the variable reflecting physician diagnosis of overweight was not time-delimited (e.g., prior 12 months), thus providing some possibility of temporal differences in the time since receipt of diagnosis. However, responses to this variable are unlikely to vary systematically by race/ethnicity. Small cell sizes in some categories may have impacted our estimates. Finally, Mexican-Americans are overrepresented in the NHANES category; thus, there should be cautioned own revelations made to other Hispanic sub-groups.

Correcting misperceptions of weight status may be necessary to actively and successfully engage individuals in overweight and obesity control efforts. As increasing attention is directed towards the treatment and prevention of obesity among racial/ethnic minorities, identifying similar disproportionately prevalent sociocultural influences should be a high priority.

## Declaration of competing interests

The authors declare that they have no competing interests.

## Authors' contributions

GB and KY equally contributed to the conception, design, acquisition of data, analysis and interpretation of data, drafting of the manuscript and have both given approval of the final version for publication.

## References

[B1] Ogden CL, Carroll MD, Curtin LR, McDowell MA, Tabak CJ, Flegal KM (2006). Prevalence of overweight and obesity in the United States, 1999-2004. Jama.

[B2] Flynn KJ, Fitzgibbon M (1998). Body images and obesity risk among black females: a review of the literature. Ann Behav Med.

[B3] Kumanyika S, Wilson JF, Guilford-Davenport M (1993). Weight-related attitudes and behaviors of black women. J Am Diet Assoc.

[B4] Striegel-Moore RH, Wilfley DE, Caldwell MB, Needham ML, Brownell KD (1996). Weight-related attitudes and behaviors of women who diet to lose weight: a comparison of black dieters and white dieters. Obes Res.

[B5] Stevens J, Kumanyika SK, Keil JE (1994). Attitudes toward body size and dieting: differences between elderly black and white women. Am J Public Health.

[B6] Smith DE, Thompson JK, Raczynski JM, Hilner JE (1999). Body image among men and women in a biracial cohort: the CARDIA Study. Int J Eat Disord.

[B7] Powell AD, Kahn AS (1995). Racial differences in women's desires to be thin. Int J Eat Disord.

[B8] Altabe M (1998). Ethnicity and body image: quantitative and qualitative analysis. Int J Eat Disord.

[B9] Kuchler F, Variyam JN (2003). Mistakes were made: misperception as a barrier to reducing overweight. Int J Obes Relat Metab Disord.

[B10] Paeratakul S, White MA, Williamson DA, Ryan DH, Bray GA (2002). Sex, race/ethnicity, socioeconomic status, and BMI in relation to self-perception of overweight. Obes Res.

[B11] Bennett GG, Wolin KY, Goodman M, Samplin-Salgado M, Carter P, Dutton S, Hill R, Emmons K (2006). Attitudes Regarding Overweight, Exercise, and Health among Blacks (United States). Cancer Causes Control.

[B12] (2003). Screening for obesity in adults: recomendations and rationale. Ann Intern Med.

[B13] (2003). Behavioral counseling in primary care to promote a healthy diet: recommendations and rationale. Am J Prev Med.

[B14] Institute of Medicine (U.S.), Subcommittee on Military Weight Management (2003). Weight management : state of the science and opportunities for military programs.

[B15] Klein S, Burke LE, Bray GA (2004). Clinical implications of obesity with specific focus on cardiovascular disease: a statement for professionals from the American Heart Association Council on Nutrition, Physical Activity, and Metabolism: endorsed by the American College of Cardiology Foundation. Circulation.

[B16] United States Public Health Service, Office of the Surgeon General, Office of Disease Prevention and Health Promotion, Centers for Disease Control and Prevention (U.S.), National Institutes of Health (U.S.) (2001). The Surgeon General's call to action to prevent and decrease overweight and obesity.

[B17] Centers for Disease Control and Prevention (CDC), National Center for Health Statistics (NCHS) (1999). National Health and Nutrition Examination Survey Data.

[B18] Centers for Disease Control and Prevention (CDC), National Center for Health Statistics (NCHS) (2001). National Health and Nutrition Examination Survey Data.

[B19] Centers for Disease Control and Prevention (CDC), National Center for Health Statistics (NCHS) (1999). National Health and Nutrition Examination Survey Mobile Examination Center Protocol.

[B20] Centers for Disease Control and Prevention (CDC), National Center for Health Statistics (NCHS) (2001). National Health and Nutrition Examination Survey Mobile Examination Center Protocol.

[B21] Centers for Disease Control and Prevention (CDC), National Center for Health Statistics (NCHS) (1999). National Health and Nutrition Examination Survey Questionnaire.

[B22] Stafford RS, Farhat JH, Misra B, Schoenfeld DA (2000). National patterns of physician activities related to obesity management. Arch Fam Med.

